# PP2A^Cdc55^’s role in reductional chromosome segregation during achiasmate meiosis in budding yeast is independent of its FEAR function

**DOI:** 10.1038/srep30397

**Published:** 2016-07-26

**Authors:** Gary W. Kerr, Jin Huei Wong, Prakash Arumugam

**Affiliations:** 1 School of Environment & Life Sciences, University of Salford, Manchester, UK; 2 Bioinformatics Institute (A*STAR), 30 Biopolis Street, #07-01 Matrix, 138671, Singapore; 3 Department of Biological Sciences, National University of Singapore, 14 Science Drive 4, 117543, Singapore.

## Abstract

PP2A^Cdc55^ is a highly conserved serine-threonine protein phosphatase that is involved in diverse cellular processes. In budding yeast, meiotic cells lacking PP2A^Cdc55^ activity undergo a premature exit from meiosis I which results in a failure to form bipolar spindles and divide nuclei. This defect is largely due to its role in negatively regulating the Cdc Fourteen Early Anaphase Release (FEAR) pathway. PP2A^Cdc55^ prevents nucleolar release of the Cdk (Cyclin-dependent kinase)-antagonising phosphatase Cdc14 by counteracting phosphorylation of the nucleolar protein Net1 by Cdk. *CDC55* was identified in a genetic screen for monopolins performed by isolating suppressors of *spo11*Δ *spo12*Δ lethality suggesting that Cdc55 might have a role in meiotic chromosome segregation. We investigated this possibility by isolating *cdc55* alleles that suppress *spo11*Δ *spo12*Δ lethality and show that this suppression is independent of PP2A^Cdc55^’s FEAR function. Although the suppressor mutations in *cdc55* affect reductional chromosome segregation in the absence of recombination, they have no effect on chromosome segregation during wild type meiosis. We suggest that Cdc55 is required for reductional chromosome segregation during achiasmate meiosis and this is independent of its FEAR function.

Meiosis is a specialised form of cell division in sexually reproducing organisms by which haploid daughter cells are produced from diploid germ cells. This is different from mitotic cycle in which daughter cells produced have the same DNA content as their parental cells^1^,^2^. Meiotic cells execute two nuclear divisions (meiosis I and II) following a single round of DNA replication. Four crucial innovations during meiosis help to achieve the remarkable feat of halving ploidy^1^,^2^. Firstly, reciprocal recombination and formation of chiasmata between homologous chromosomes occur during prophase I. Secondly, sister kinetochores are mono-oriented on the meiosis I spindle. Thirdly, centromeric cohesion is protected during meiosis I i.e., cohesion is destroyed distal (but not proximal) to chiasmata. Cohesion is preserved around centromeres until meiosis II to help dyad chromosomes to bi-orient on the meiosis II spindle. Finally, DNA replication is inhibited between meiosis I and meiosis II.

Insights into the mechanism of monopolar attachment have largely come from research with budding yeast (*Saccharomyces cerevisiae*) and fission yeast (*Schizosaccharomyces pombe*). In *S. cerevisiae*, monopolar attachment is dependent on monopolin complex which consists of four subunits namely Mam1, Csm1, Lrs4 and Hrr25. Cells lacking any of these components bi-orient sister centromeres during meiosis I and attempt to separate sister chromatids^3^,^4^,^5^. While Mam1 is meiosis-specific^3^, the casein kinase Hrr25 and nucleolar proteins Csm1 and Lrs4 are expressed during both mitosis and meiosis I & II^4^,^5^. Csm1 and Lrs4 remain in the nucleolus during most of mitotic cell cycle and are released only during meiosis I by polo kinase Cdc5^4^,^6^. Following nucleolar release, Lrs4 is hyperphosphorylated by Dbf4-dependent kinase Cdc7 and Cdc5/Spo13^7^. Hyperphosphorylation of Lrs4 promotes monopolin’s association with kinetochores. Monopolin’s binding to kinetochores and function is dependent on a physical interaction between Csm1 and Dsn1, a component of the MIND complex^8^,^9^.

PP2A belongs to a highly conserved family of serine-threonine phosphatases involved in diverse cellular processes. PP2A phosphatase consists of 3 subunits-a catalytic subunit (Pph21/Pph22), a scaffold subunit (Tpd3) and a regulatory subunit (Rts1/Cdc55). PP2A^Cdc55^ refers to a form that contains Pph21/22, Tpd3 and Cdc55. *CDC55* gene was isolated in a genetic screen for monopolin genes^4^. However the extremely poor growth of *cdc55*Δ cells precluded analysis of PP2A^Cdc55^’s role in monopolar attachment. Phenotypic analyses of strains bearing a meiotic null allele of *CDC55* (*P*_*CLB2*_*-CDC55*) revealed that PP2A^Cdc55^ is required for preventing premature exit from meiosis I^10^,^11^. During mitosis and meiosis I, PP2A^Cdc55^ prevents Cdc14 release from the nucleous by counteracting phosphorylation of Net1 by Cdk^10^,^11^,^12^. During meiosis, *P*_*CLB2*_*-CDC55* cells release Cdc14 prematurely from the nucleolus, fail to form bipolar spindles and do not undergo meiotic divisions^10^. A form of Net1 that lacks 6 Cdk phosphorylation sites (*net1-6Cdk*) suppresses the nuclear division defect of *P*_*CLB2*_*-CDC55* cells^10^. However the spore viability of *net1-6Cdk P*_*CLB2*_*-CDC55* cells is reduced compared to *NET1* and *net1-6Cdk* cells^10^, leaving open the possibility that PP2A^Cdc55^ might play a role in meiotic chromosome segregation.

To assess PP2A^Cdc55^’s role in meiotic chromosome segregation, we performed a genetic screen and isolated two classes of suppressor mutations within the *CDC55* ORF. Class A mutants suppressed the dyad phenotype of *spo11*Δ *spo12*Δ strains. Class B mutants rescued the poor spore viability phenotype of *spo11*Δ *spo12*Δ strains. We show that the Class A mutations affect the FEAR function of Cdc55 confirming the antagonistic roles of Cdc55 and Spo12 in the FEAR pathway. Furthermore, we demonstrate that the Class B mutations affect reductional segregation of sister centromeres during achiasmate meiosis but not during wild type meiosis. We suggest that PP2A^Cdc55^ is required for reductional chromosome segregation in the absence of recombination and that this function is distinct from its role in the FEAR pathway.

## Results

### Mutations in *CDC55* suppress the poor spore viability and dyad phenotype of *spo11*Δ *spo12*Δ strains

**Figure 1:**
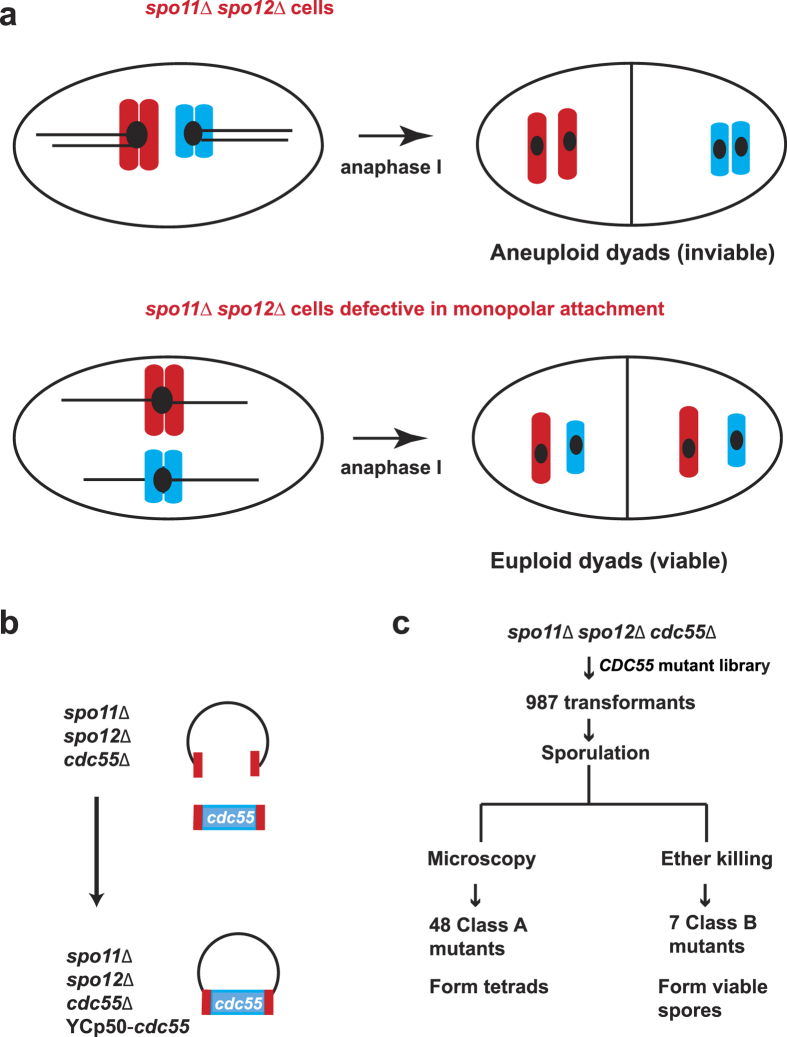
Isolation of ‘monopolin’ alleles of *cdc55*. (**a**) *spo11*Δ *spo12*Δ cells form inviable aneuploid dyads due to reductional segregation of sister chromatids during anaphase I (top panel). Disabling monopolar attachment in *spo11*Δ *spo12*Δ cells results in equational segregation of sister chromatids and production of viable euploid dyads (bottom panel). Anaphase I segregation of two pairs of non-homologous sister chromatids (in red and blue) with kinetochores (as black circles) and spindle (as a black line) is depicted in the schematic. (**b**) *spo11*Δ *spo12*Δ *cdc55*Δ cells were transformed using with a library of *cdc55* mutant alleles generated by random PCR mutagenesis followed by gap-repair mediated transformation. (**c**) Screen generated two classes of mutants. Class A mutants suppressed the *spo12*Δ dyad phenotype and were identified by dark-field microscopy. Class B mutants (monopolin) produced viable spores as determined by performing the ether-killing based spore viability assay.

The inability of *P*_*CLB2*_*-CDC55* cells to form tetrads is partially suppressed by *net1-6Cdk*^10^. However the spore viability of *P*_*CLB2*_*-CDC55 net1-6Cdk* cells was around 50% compared to around 90% for *NET1* and *net1-6Cdk* cells^10^. Reduced spore viability could be due to incomplete suppression of *P*_*CLB2*_*-CDC55* by *net1-6Cdk*. Alternatively, PP2A^Cdc55^ may have a role in accurate meiotic chromosome segregation that is distinct from its FEAR function. Interestingly, *CDC55* was isolated in a genetic screen for monopolin mutants^4^. To determine Cdc55’s role in meiotic chromosome segregation, we isolated ‘monopolin’ alleles of *CDC55*. We targeted the *CDC55* open reading frame (ORF) to PCR-based mutagenesis and selected for mutants that suppress the low spore viability of the *spo11*Δ *spo12*Δ diploids. Deletion of both *SPO11* and *SPO12* is marked by severely low spore viability due to co-segregation of sister centromeres without bi-orientation of homologues^4^. *SPO11* is a meiosis-specific gene that initiates meiotic recombination by catalysing the formation of DNA double-strand breaks^13^. Spo12 is a nucleolar protein required for Cdc14 for exit from meiosis I and its absence during meiosis results in production of dyads^14^. Inactivating monopolar attachment in a *spo11*Δ *spo12*Δ genetic background causes sister centromeres to segregate equationally and increases spore viability (Fig. 1a)^4^. A library of *CDC55* mutant alleles was generated by random PCR mutagenesis followed by gap-repair in *spo11*Δ *spo12*Δ *cdc55*Δ cells (Fig. 1b). The transformants were induced to sporulate by transferring them to Sporulation Medium (SPM). Microscopic examination of spores surprisingly revealed generation of mutations that suppressed the dyad phenotype of *spo11*Δ *spo12*Δ strains i.e. the mutants formed tetrads. Transformants with high spore viability were selected using the ether-killing assay^15^. Exposure to ether vapours preferentially kills vegetative cells but not spores. This genetic screen thus identified two classes of *cdc55* mutants (Fig. 1c). Class A- These suppressed the dyad but not the spore inviability phenotype of *spo11*Δ *spo12*Δ strains. Class B- they suppressed the spore inviability phenotype of *spo11*Δ *spo12*Δ strains.

### Suppressors of *spo12*Δ dyad phenotype

**Figure 2:**
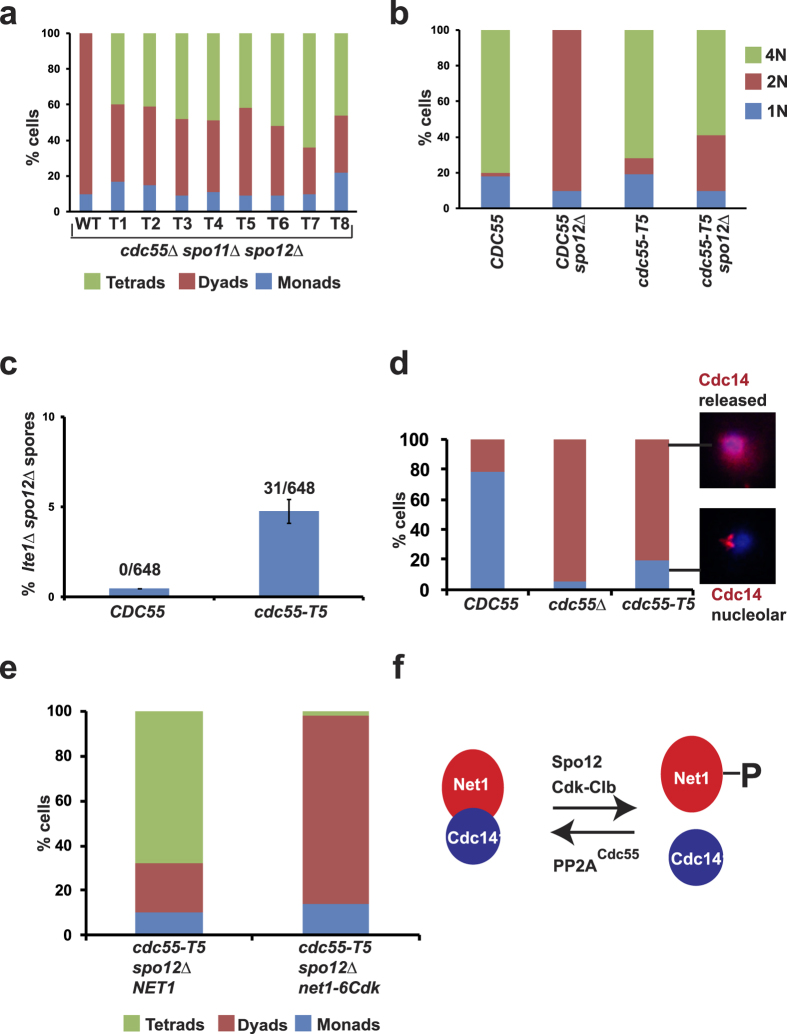
*cdc55-T5* mutants release Cdc14 prematurely from the nucleolus. (**a**) Class A mutations in *cdc55* partially suppress the *spo11*Δ *spo12*Δ dyad phenotype. Eight alleles that suppressed dyad phenotype are labelled T1-8 (T = tetrad-forming allele) (N = 100). (**b**) *cdc55-T5* suppresses the nuclear division defect of *spo12*Δ cells. *CDC55 SPO12*, *CDC55 spo12*Δ, *cdc55-T5 SPO12*, *cdc55-T5 spo12*Δ cells were induced to sporulate by transferring them to SPM and incubating for 12 hours in a shaker at 30 °C. Cells were stained with DAPI and classified as either mononucleate, binucleate or tri/tetra-nucleate (N = 100). (**c**) Frequency of *lte1*Δ *spo12*Δ haploids produced from sporulation followed by tetrad dissection (216 tetrads each in triplicates) and germination of *LTE1/lte1*Δ *SPO12/spo12*Δ diploids carrying either *CDC55* or *cdc55-T5*. Difference in the frequencies between *CDC55* and *cdc55-T5* strains is statistically significant (Student’s t-test, P < 0.01). (**d**) *P*_*MET3*_*-CDC20* cells carrying either *CDC55* or *cdc55*Δ or *cdc55-T5* were transferred to SD medium in the presence of methionine and incubated for 160 minutes. Cells were fixed and subjected to immunostaining with anti-Cdc14 antibodies. DNA was visualized by staining with DAPI. Percentage of cells with nucleolar Cdc14 and released Cdc14 signals were calculated (N = 100). Representative images of cells with nucleolar and released Cdc14 signals are shown. (**e**) *cdc55-T5 NET1 spo12*Δ and *cdc55-T5 net1-6Cdk spo12*Δ strains were induced to sporulate by transferring them to SPM followed by incubation for 24 h. Spores were harvested and nuclear division was scored by staining with DAPI (N = 100). (**f**) Antagonistic roles of PP2A^Cdc55^ and Spo12 in the regulation of FEAR pathway.

To test whether the suppression of dyad phenotype of *spo11*Δ *spo12*Δ was due to mutations within the *CDC55* gene, the plasmids bearing *CDC55* genes were recovered from the mutants and re-introduced into the parental *spo11*Δ *spo12*Δ *cdc55*Δ strain. Eight alleles (*T1-T8*) were selected that produced the highest count of tetrads. Figure 2a shows the composition of asci from diploid *spo11*Δ *spo12*Δ *cdc55*Δ strains with these 8 alleles. While *spo11*Δ *spo12*Δ *cdc55*Δ cells transformed with a plasmid encoding wild-type *CDC55* produced 100% dyads like *spo12*Δ strains, transformants with the eight mutant alleles formed tetrads at frequencies ranging from 40–62% (Fig. 2a). Representative images of asci formed by *spo11*Δ *spo12*Δ cells carrying either wild type *CDC55* or the tetrad-forming allele of *CDC55* (*T5*) are shown in the Supplementary Figure S1. Plasmids bearing mutant *cdc55* alleles were isolated from these strains and sequenced to identify mutations that caused suppression of the dyad phenotype. The effect of mutations identified in the 8 alleles on the Cdc55 a.a. sequence are shown in Table 1.

### c*dc55-T5* suppresses the nuclear division defect of *spo12*Δ strains

We chose the *cdc55-T5* for further analysis as this was consistently the best suppressor of *spo11*Δ *spo12*Δ dyad phenotype. We integrated the *cdc55-T5* allele at its endogenous locus by homologous recombination. To test whether *cdc55-T5* suppresses the nuclear division defect of *spo12*Δ, the *CDC55 spo12*Δ and *cdc55-T5 spo12*Δ strains were induced to sporulate by transferring them to SPM. While *CDC55 spo12*Δ formed 90% binucleates but no tri/tetra-nucleates after 11 hours in SPM, the *cdc55-T5 spo12*Δ cells formed 60% tri/tetra-nucleates (Fig. 2b).

### c*dc55-T5* suppresses *spo12*Δ *lte1*Δ lethality

*cdc55-T5* suppression of *spo12*Δ dyad phenotype can be explained on basis of opposing roles of PP2A^Cdc55^ and Spo12 in FEAR regulation (Fig. 2f)^16^. Spo12 is a positive regulator of the FEAR pathway. Following Cdk-mediated phosphorylation, Spo12 helps in dissociation of the Replication Fork Barrier protein (Fob1) which stabilizes Net1-Cdc14 interaction^17^,^18^. On the other hand, PP2A^Cdc55^ has an antagonistic role in the FEAR pathway^12^. It opposes Net1 phosphorylation by Cdk during metaphase and prevents Cdc14 release from the nucleolus^12^. Therefore the inability of *spo12*Δ cells to exit from meiosis I due to the lack of Cdc14 release might be suppressed by *cdc55* mutations. If this idea were to be true one would predict the *cdc55-T5* strains to be defective in preventing Cdc14 release from the nucleolus.

We first tested whether *cdc55-T5* suppressed the synthetic lethality of *spo12*Δ and *lte1*Δ strains. Lte1 is part of the mitotic exit network (MEN) that causes a complete release of Cdc14 release during anaphase^19^,^20^. Synthetic lethality of *spo12*Δ and *lte1*Δ strains is due to the failure of *spo12*Δ *lte1*Δ strains to release Cdc14 from the nucleolus and exit from mitosis. Thus any mutation that increases Cdc14 release is expected to suppress the lethality of *spo12*Δ *lte1*Δ strains. We therefore mated MATa *lte1*Δ strains with MATα *spo12*Δ strains that contained either *CDC55* or *cdc55-T5* alleles. The diploids thus generated were sporulated. Hundred spores were dissected onto rich medium plates and haploids from viable spores were genotyped for segregation of *lte1*Δ, *spo12*Δ and *CDC55/cdc55-T5* markers. As expected, we failed to obtain any *lte1*Δ *spo12*Δ *CDC55* cells (frequency < 0.46%) indicating that *spo12*Δ is synthetic lethal with *lte1*Δ. In contrast, we obtained *cdc55-T5 lte1*Δ *spo12*Δ strains (frequency = 4.77%) indicating that *cdc55-T5* suppresses *spo12*Δ *lte1*Δ lethality and causes increased release of Cdc14 from the nucleolus (Fig. 2c).

### *cdc55-T5* cells release Cdc14 prematurely from the nucleolus

To directly test whether *cdc55-T5* cells are defective in preventing Cdc14 release from the nucleolus we constructed *P*_*MET3*_*-CDC20* strains containing either wild type *CDC55* or *cdc55-T5* or *cdc55*Δ. Cdc20, an activator of APC (Anaphase Promoting Complex), can be depleted from *P*_*MET3*_*-CDC20* strains by addition of methionine to the medium. After 160 minutes of incubation of the 3 strains in SD medium containing methionine, Cdc14 localization was detected by immunostaining with anti-Cdc14 antibodies. In *CDC55* cells, only 18% of cells had Cdc14 released from the nucleolus. In contrast, about 95% of *cdc55*Δ cells and 81% of *cdc55-T5* cells released Cdc14 from the nucleolus (Fig. 2d). If enhanced phosphorylation of Net1 by Cdk in *cdc55-T5* cells was responsible for suppression of *spo12*Δ dyad phenotype, then *net1-6Cdk* should negate the suppression. To test this we generated *cdc55-T5 spo12*Δ diploid strains with wild type *NET1* or *net1-6Cdk*. While *cdc55-T5 spo12*Δ *NET1* strains formed around 68% tetrads and 22% dyads, *cdc55-T5 spo12*Δ *net1-6Cdk cells* formed 84% dyads and 2% tetrads (Fig. 2e). Taken together, our results indicate that mutations in *CDC55* that suppress *spo12*Δ dyad phenotype are defective in restraining Cdc14 in the nucleolus.

### Class B mutants -monopolin alleles of *CDC55*

**Figure 3:**
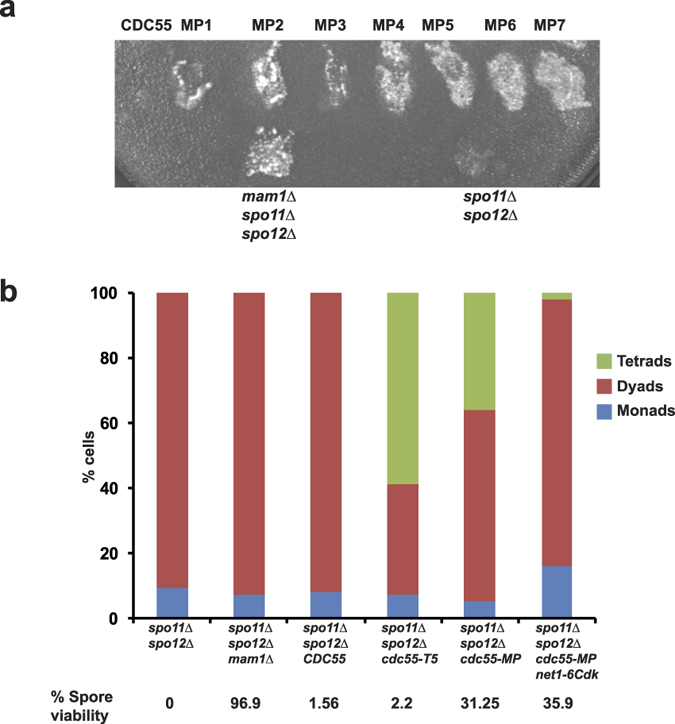
PP2A^Cdc55^’s role in monopolar attachment is genetically separable from its role in meiotic FEAR regulation. (**a**) *spo11*Δ *spo12*Δ *cdc55*Δ cells carrying plasmids encoding either wild type *CDC55* or class B mutant *cdc55* alleles (MP1-7) were sporulated. Spore viability was assessed using the ether-killing assay. *spo11*Δ *spo12*Δ and *spo11*Δ *spo12*Δ *mam1*Δ strains served as negative and positive controls respectively. (**b**) *spo11*Δ *spo12*Δ cells and *spo11*Δ *spo12*Δ strains carrying either *mam1*Δ or *CDC55* or *cdc55-T5* or *cdc55-MP* or *cdc55-MP net1-6Cdk* were induced to sporulate by transferring them to SPM followed by incubation for 24 h. Spores were harvested and nuclear division was scored by staining with DAPI (N = 100). Spore viability was determined by dissecting dyads onto YPD plates and grown at 30 °C. Spore viability (n = 100) was scored after 3 days.

In contrast to Class A mutants, Class B mutants partially suppressed the spore inviability phenotype of *spo11*Δ *spo12*Δ strains. They also suppressed the dyad phenotype of *spo11*Δ *spo12*Δ strains to variable extents. This suggests that the class B mutations affect a function of PP2A^Cdc55^ that is distinct from its role in the FEAR pathway. In the genetic screen described above, we obtained seventeen class B mutants that had high spore viability (Fig. 1c). In order to confirm whether the high spore viability phenotype was caused due to mutations within the *CDC55* ORF, plasmids were isolated from the 17 mutant strains and re-introduced into the parent *spo11*Δ *spo12*Δ *cdc55*Δ strain. This secondary screen confirmed the high spore viability phenotype of 7 alleles named *cdc55-MP1 to cdc55-MP7* (Fig. 3a). Primers designed across the *CDC55* open reading frame (ORF) were used to sequence the mutations within the 7 alleles. The effect of these mutations on Cdc55 a.a. sequence is presented in Table 2. To maximize the severity of the phenotype we combined the *cdc55-MP1* and *cdc55-MP4* mutations to create an allele *cdc55-MP* which we used for further analysis.

### Suppression of *spo11*Δ *spo12*Δ lethality by *cdc55-MP* is independent of PP2A^Cdc55^’s FEAR function

To determine whether impairing Cdc55’s FEAR function suppresses the *spo11*Δ *spo12*Δ lethality, we tested the ability of *cdc55-T5* to suppress *spo11*Δ *spo12*Δ lethality. The spore viabilities of dyads produced by *CDC55 spo11*Δ *spo12*Δ and *cdc55-T5 spo11*Δ *spo12*Δ cells were 2 and 4% respectively compared to 31% for *cdc55-MP spo11*Δ *spo12*Δ cells (Fig. 3b). Tetrads obtained from *cdc55-MP* cells had low spore viability (4.2%), which is expected as the second meiotic division will be random in these cells. Since the *cdc55-T5* mutant is defective in preventing Cdc14 release (see above), our results suggest that a defect in Cdc55’s FEAR function *per se* is not sufficient to rescue the poor spore viability of *spo11*Δ *spo12*Δ strains. However the *cdc55-MP* allele also partially suppressed *spo12*Δ dyad phenotype (Fig. 3a). This leaves open the possibility that a FEAR defect in Cdc55 might contribute to suppression of *spo11*Δ *spo12*Δ spore inviability phenotype. Since *net1-6Cdk* suppresses the nuclear division defect of *P*_*CLB2*_*-CDC55* cells^10^ and *cdc55-T5* cells (see above), we generated *cdc55-MP spo11*Δ *spo12*Δ *net1-6Cdk* strains to overcome the FEAR defect of *cdc55-MP* cells. Indeed, *cdc55-MP spo11*Δ *spo12*Δ *net1-6Cdk* cells formed 100% dyads suggesting that *net1-6Cdk* suppressed the FEAR defect of *cdc55-MP* cells (Fig. 3a). However the spore viability of *cdc55-MP spo11*Δ *spo12*Δ *net1-6Cdk* cells was still high (35%) indicating that the FEAR defect in *cdc55-MP* cells does not contribute to suppression of *spo11*Δ *spo12*Δ spore lethality phenotype (Fig. 3b).

### *cdc55-MP* affects reductional chromosome segregation during achiasmate meiosis

**Figure 4:**
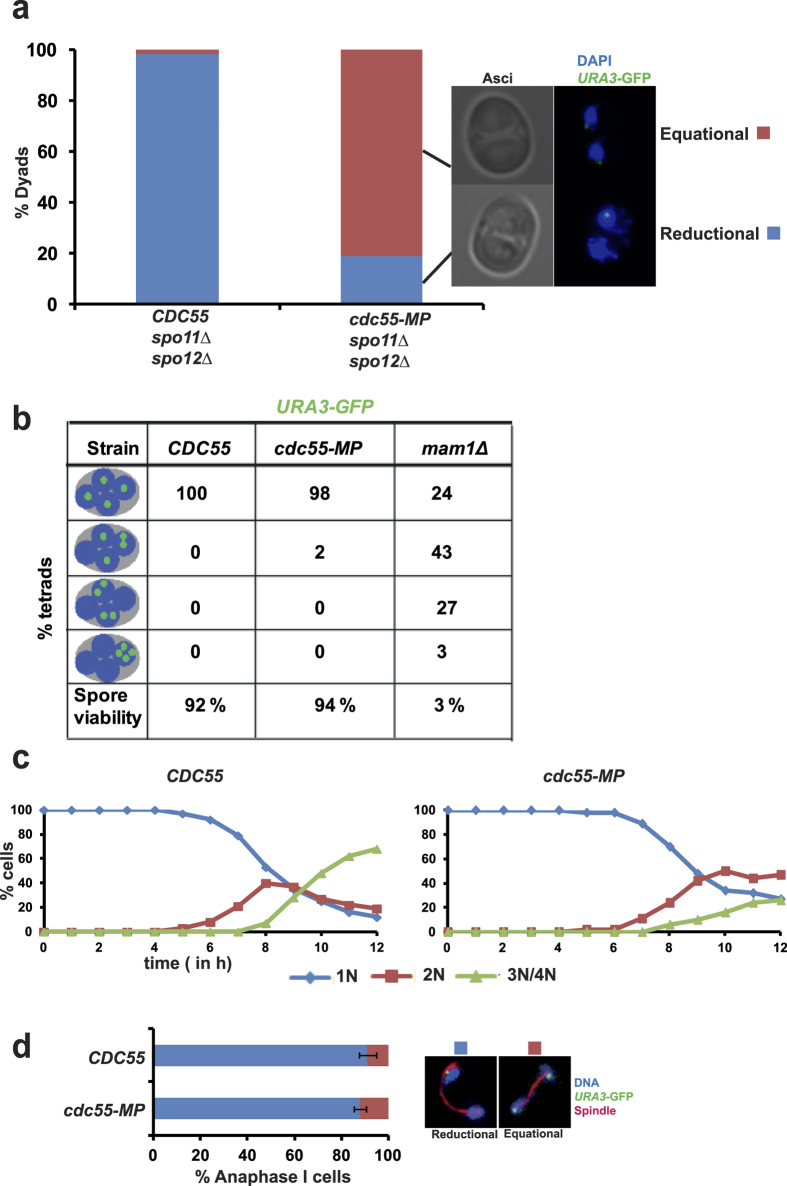
*cdc55-MP* affects reductional segregation of chromosomes during meiosis I in *spo11*Δ *spo12*Δ cells but not in wild type cells. (**a**) *CDC55 spo11*Δ *spo12*Δ and *cdc55-MP spo11*Δ *spo12*Δ cells containing heterozygous GFP-tagged *URA3* sequences were induced to enter meiosis by transferring them into SPM followed by incubation at 30 °C for 24 h. Cells were fixed and stained with DAPI to visualize DNA and segregation of GFP-tagged *URA3* dots was assayed by fluorescence microscopy (N = 200). Representative images of dyads showing equational and reductional segregation of *URA3*-GFP sequences are depicted on the right. (**b**) Detection of homozygous *URA3*-GFP and DNA in tetrads produced by *CDC55*, *cdc55-MP* and *mam1*Δ strains. Tetrads produced by the strains were dissected onto YPD plates and grown at 30 °C. Spore viability (n = 100) was scored after 3 days. (**c**,**d**) Analysis of meiosis in *CDC55* and *cdc55-MP* cells containing heterozygous *URA3*-GFP. Samples of cultures at indicated time points were taken out, fixed and stained with DAPI and anti-tubulin antibodies. (**c**) Kinetics of meiotic nuclear division shown by percentage of mononucleate, binucleate and tri/tetranucleate cells during the time course. (**d**) Proportion of cells containing anaphase I spindles undergoing reductional and equational segregation of *URA3*-GFP dots are depicted (N = 100 in triplicates). Difference in the frequencies of reductional segregation between *CDC55* and *cdc55-MP* strains is not statistically significant (Student’s t-test, P < 0.1). Data shown are representative of 3 independent experiments.

We investigated whether *cdc55-MP* affects reductional segregation of chromosomes in *spo11*Δ *spo12*Δ cells by analyzing segregation of sister centromeres in *CDC55 spo11*Δ *spo12*Δ and *cdc55-MP spo11*Δ *spo12*Δ cells. To visualize chromosome segregation, we tagged the *URA3* locus (located 30 kb from the centromere) in one of the two parental chromosome V’s with GFP using the tetO/TetR system. *CDC55 spo11*Δ *spo12*Δ and *cdc55-MP spo11*Δ *spo12*Δ cells were induced to enter meiosis by transferring them into SPM. In the absence of recombination, homologs are not connected and segregate randomly. However sister centromeres are mono-oriented and move towards the same spindle pole. Consistent with this, in *CDC55 spo11*Δ *spo12*Δ cells, about 97% of *URA3*-GFP dots segregated reductionally (Fig. 4a). In contrast, about 80% of *URA3*-GFP dots segregated equationally in *cdc55-MP spo11*Δ *spo12*Δ cells (Fig. 4a). This suggests that PP2A^Cdc55^ is required for reductional segregation of sister chromatids during achiasmate meiosis.

### *cdc55-MP* does not affect chromosome segregation during wild type meiosis

We then tested whether *cdc55-MP* affects chromosome segregation during wild type meiosis. We monitored chromosome segregation in *CDC55* and *cdc55-MP* cells by tagging homologous chromosomes with GFP and observing their segregation into tetranucleate cells. We used *mam1*Δ cells as a control. While *mam1*Δ cells missegregated chromosomes and produced inviable spores, *CDC55* and *cdc55-MP* cells segregated chromosomes normally and produced viable spores (Fig. 4b). We also monitored the kinetics of nuclear division and segregation of GFP-tagged *URA3* dots in wild type and *cdc55-MP* cells during meiosis I. *cdc55-MP* cells underwent 2 rounds of nuclear division albeit with somewhat delayed kinetics compared to wild type cells (Fig. 4c). However, there was no difference in the extent of reductional segregation of *URA3*-GFP dots between wild type and *cdc55-MP* cells (Fig. 4d). Taken together our results suggest that *cdc55-MP* has no major effect on chromosome segregation during wild type meiosis.

### Ability of *cdc55-MP* to suppress *spo11*Δ *spo12*Δ spore lethality is recessive and not due to premature Clb3 expression or spindle checkpoint function

**Figure 5:**
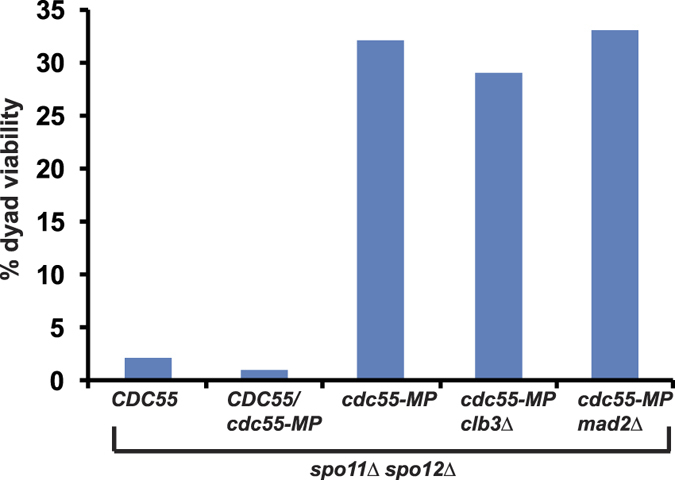
Increased spore viability of *cdc55-MP spo11*Δ *spo12*Δ cells is recessive and is not due to premature Clb3 expression or checkpoint function. Dyads produced by *spo11*Δ *spo12*Δ *CDC55*, *spo11*Δ *spo12*Δ *cdc55-MP*, *spo11*Δ *spo12*Δ *CDC55/cdc55-MP*, *spo11*Δ *spo12*Δ *cdc55-MP clb3*Δ and *spo11*Δ *spo12*Δ *cdc55-MP mad2*Δ strains were dissected on YPD plates and grown at 30 °C. Spore viability (n = 100) was scored after 3 days.

Cyclin Clb3 is transcribed during meiosis I but translated during meiosis II^21^. Expression of Clb3 during meiosis I affects monopolar attachment and protection of centromeric cohesion^21^. To test whether premature expression of Clb3 could cause the reductional chromosome defect of *cdc55-MP spo11*Δ cells we tested the effect of *clb3*Δ on the ability of *cdc55-MP* to suppress the poor spore viability phenotype of *spo11*Δ *spo12*Δ strains. Both *spo11*Δ *spo12*Δ *cdc55-MP clb3*Δ and *spo11*Δ *spo12*Δ *cdc55-MP CLB3* strains had comparable spore viabilities indicating that ability of *cdc55-MP* to suppress *spo11*Δ *spo12*Δ is not due to premature expression of Clb3 during meiosis (Fig. 5). In the absence of recombination, sister centromeres segregate towards the same spindle pole, although the sister kinetochores are not under tension. This suggests that the meiotic checkpoint machinery does not correct such kinetochore-microtubule connections. It was formally possible that the inability of the spindle checkpoint to correct monopolar attachments was dependent on PP2A^Cdc55^ and *cdc55-MP* affected this process. However, deletion of the gene encoding the Spindle Checkpoint component *MAD2* had no effect on the ability of *cdc55-MP* to suppress *spo11*Δ *spo12*Δ lethality (Fig. 5). We also ruled out the possibility that *cdc55-MP* was a gain-of-function mutation as its effect on *spo11*Δ *spo12*Δ spore lethality was recessive (Fig. 5).

## Discussion

PP2A^Cdc55^ is a highly conserved protein phosphatase that is involved in diverse biological processes. We previously reported that PP2A^Cdc55^ is required for preventing premature exit from meiosis I by counteracting phosphorylation of Net1 by Cdk. However it left open the possibility that PP2A^Cdc55^ might have an additional role in meiotic chromosome segregation. By targeting the *CDC55* ORF to PCR mutagenesis, we isolated mutant *cdc55* alleles that either suppressed the *spo12*Δ dyad phenotype or suppressed *spo11*Δ *spo12*Δ lethality.

Suppressors of *spo12*Δ dyad phenotype are defective in restraining Cdc14 in the nucleolus during metaphase and confirm the antagonistic roles of Spo12 and PP2A^Cdc55^ in the FEAR pathway. Spo12 mutants fail to release Cdc14 from the nucleolus resulting in an inability to undergo exit from meiosis I and consequently form dyads^22^,^23^. On the other hand, meiotic cells lacking PP2A^Cdc55^ release Cdc14 prematurely from the nucleolus, undergo precocious exit from meiosis I and form monads^10^. We suggest that Class A mutations are hypomorphic alleles of *cdc55* that result in Cdc14 release that is sufficient to rescue the *spo12*Δ dyad phenotype but not high enough to cause a premature exit from meiosis I. It will be interesting to test whether Class A mutations affect interaction of PP2A^Cdc55^ with Net1, its putative substrate in the FEAR pathway.

Suppressors of *spo11*Δ *spo12*Δ lethality affect reductional chromosome segregation in recombination-deficient cells but had no noticeable effect in recombination-proficient cells. We further demonstrate that suppression of *spo11*Δ *spo12*Δ spore lethality is not due to loss of PP2A^Cdc55^’s FEAR function. It has been reported that chiasmata promotes efficient monopolar attachment of sister kinetochores during meiosis I in fission yeast cells^24^,^25^. Although, chiasmata in budding yeast are dispensable for monopolar attachment^3^, they may modestly promote sister kinetochore co-orientation, which is not apparent in wild type cells but detectable in *cdc55-MP* cells. It is also possible that *cdc55-MP* allele is defective in pericentromeric cohesion which may facilitate bi-orientation of sister centromeres during achiasmate meiosis, However the presence of chiasmata might suppress this defect in *cdc55-MP* cells possibly by physically constraining the sister kinetochores to face the same spindle pole or by creating tension across centromeric regions. Comparing the binding partners of *CDC55* and *cdc55-MP* encoded proteins and phosphorylation status of monopolin (and related proteins) during meiosis in *CDC55* and *cdc55-MP* strains will be informative. In male *Drosophila melanogaster*, meiosis takes place without any recombination^26^. As PP2A^Cdc55^ is a highly conserved phosphatase, it would be interesting to test whether it has any role during achiasmate meiosis in *Drosophila* males.

## Methods

All strains used are derivatives of the SK1 strain and are indicated in Table S1. Meiotic cultures and *in situ* analysis were performed as described previously^9^,^10^. YCp50 plasmid encoding *CDC55* was a gift from Prof. Tamar Kleinberger^27^. Mutagenic PCR followed by gap-repair mediated transformation was performed as described earlier^5^. Details of primers used for mutagenic PCR are available upon request.

### Spore viability assay by ether killing

Diploid cells were grown on a YPG plate (YEP+ 2% glycerol). After 24 hours, the cells were transferred to a YPD plate. After another 24 hours, cells were transferred on to a SpoVB plate and incubated for 48 hours. Cells were then replica plated onto a large glass plate containing YPD + 0.1M (NH_4_)_2_SO_4_. Thick filter paper (Whatman 2MM) was soaked in ether and placed on the inner part of the lid of the glass plate. The lid was put on top of the plate and plates were kept in a closed chamber, containing a beaker filled with ether, for 40 minutes, with the filter paper being re-soaked in ether after 20 minutes. Lids and filter paper were then removed from the plates and plates were allowed to air dry in the fume hood for 1 h. Plates were then incubated at 30 °C for 14–18 h until spore viability could be assayed.

### Immunostaining

Monoclonal rat anti–α-tubulin (1:500; AbD Serotec) and rabbit anti-Cdc14 antibody (SC-33628; Santa Cruz Biotechnology, Inc.), Secondary antibodies, pre-absorbed against sera from other species used in labeling were conjugated with Cy3 (Millipore) or Alexa Fluor 488 (Invitrogen) and diluted 1:500 (Cy3 and Alexa Fluor 488). DNA was visualized by staining with DAPI.

## Additional Information

**How to cite this article**: Kerr, G. W. *et al*. PP2A^Cdc55^’s role in reductional chromosome segregation during achiasmate meiosis in budding yeast is independent of its FEAR function. *Sci. Rep.* **6**, 30397; doi: 10.1038/srep30397 (2016).

## Supplementary Material

Supplementary Information

## Figures and Tables

**Table 1:** Effect of Class A mutations on Cdc55 a.a. sequence.

*cdc55-T2*	K60E, N71I, D493V
*cdc55-T3*	K9E, S422N
*cdc55-T4*	K57S
*cdc55-T5*	M365K, N378S
*cdc55-T6*	I307V, F521L
*cdc55-T7*	D107V, T109A, C261R, S341G, I349F, M374V, Q466P
*cdc55-T8*	K114N, R473G

**Table 2:** Effect of Class B mutations on Cdc55 a.a. sequence.

*cdc55-MP1*	K405N
*cdc55-MP2*	L520S
*cdc55-MP3*	F64S
*cdc55-MP4*	Q12P, C60Y, N144T
*cdc55-MP5*	V132E
*cdc55-MP6*	I28F, A481E
*cdc55-MP7*	I190V, S252P
