# Supplementary material for: PP2ACdc55’s role in reductional chromosome segregation during achiasmate meiosis in budding yeast is independent of its FEAR function
Source: Sci Rep. 2016 Jul 26;6:30397. doi: 10.1038/srep30397 (PMC4960654; doi:10.1038/srep30397)
Supplement: Supplementary Information [file srep30397-s1.pdf]

PP2A^{Cdc55}'s role in reductional chromosome segregation during achiasmate meiosis in budding yeast is independent of its FEAR function

Gary W. Kerr[1] , Jin Huei Wong[2] and Prakash Arumugam[2, 3]*

1. University of Salford, UK.
2. Bioinformatics Institute, Singapore 138673
3. National University of Singapore, Department of Biological Sciences,

***Corresponding author Email**: prakasha@bii.a-star.edu.sg

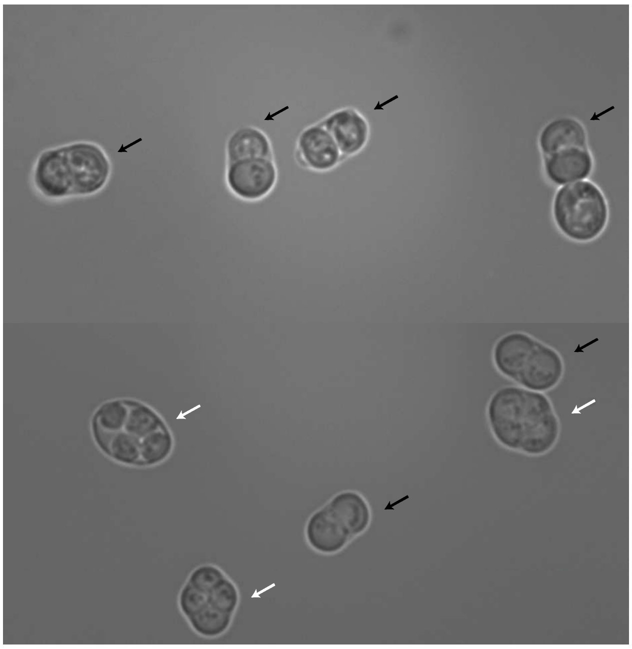

Supplementary Figure 1
***cdc55-T5* partially suppresses the dyad phenotype of *spo11Δ spo12Δ* cells.**

spo11Δ spo12Δ cells containing either *CDC55* or *cdc55-T5* were sporulated and the resulting asci were examined by bright-field microscopy. Tetrads and dyads are indicated by white and dark arrows respectively.

Supplementary Table 1 List of yeast strains used in the paper.

All yeast strains used are derivatives of SK1 and have the following markers, unless otherwise stated stated. *ho::LYS2/ho::LYS2, ura3/ura3, leu2::hisG/leu2::hisG, trp1::hisG/trp1::hisG, his3::hisG/his3::hisG, lys2/lys2.* Strains used for experiments described in the figures are listed in the table below. All markers are homozygous in diploid strains unless mentioned otherwise.

**Strain name**	**Genotype**	**Used in Figure(s)**
Y1707	MATa/α *cdc55Δ::natMX4 spo11Δ::TRP1 (K. lactis) spo12Δ::KanMX4*	2a
Y1905	MATa/α *cdc55:: CDC55-hphMX6::natMX4 P_{URA3}::tetR::GFP-LEU2, rec8:REC8-ha3::URA3, PDS1-myc18::TRP1(K.lactis) ura3/ura3:tetOx224-URA3*	2b
Y1906	MATa/α *cdc55:: CDC55-hphMX6::natMX4 spo12:KanMX6 P_{URA3}::tetR::GFP-LEU2, rec8:REC8-ha3::URA3, PDS1-myc18::TRP1(K.lactis) ura3/ura3:tetOx224-URA3*	2b
Y1907	MATa/α *cdc55:: cdc55-T5-hphMX6::natMX4 P_{URA3}::tetR::GFP-LEU2 rec8:REC8-ha3::URA3 PDS1-myc18::TRP1(K.lactis) ura3/ura3:tetOx224-URA3*	2b
Y1908	MATa/α *cdc55:: cdc55-T5-hphMX6::natMX4 spo12:KanMX4 P_{URA3}::tetR::GFP-LEU2 rec8:REC8-ha3::URA3 PDS1-myc18::TRP1(K.lactis) ura3/ura3:tetOx224-URA3*	2b
Y1994	MATa *lte1Δ::HIS3MX6*	2c

Y1998	MATα *cdc55:: CDC55-hphMX6::natMX4 spo12:KanMX4*	2c
Y2000	MATα *cdc55:: cdc55-T5-hphMX6::natMX4 spo12Δ::KanMX4*	2c
Y1991	MATa *cdc55Δ-::natMX4 NET1::ha3:HIS3 cdc20::*P_{MET3}*-CDC20::TRP1*	2d
Y1992	MATa *cdc55::CDC55::natMX4 NET1::ha3:HIS3 cdc20::*P_{MET3}*-CDC20::TRP1*	2d
Y1993	MATa *cdc55::cdc55-T5-hphMX6::natMX4 NET1::ha3-HIS3 cdc20::* P_{MET3}*-CDC20::TRP1*	2d
Y2154	MATa/α *spo12Δ::KanMX4 cdc55Δ::cdc55-T5-hphMX6::natMX4 net1Δ::his5, NET1--TEVmyc9::TRP1*	2e
Y2155	MATa/α *spo12Δ::KanMX4 cdc55Δ:: cdc55-T5-hphMX6::natMX4 net1Δ::his5, net1-6CDK-TEVmyc9::TRP1*	2e
Y1018	MATa/α *spo11Δ::TRP1 (K. lactis) spo12Δ::KanMX4*	3a and b
Y1019	MATa/α *spo11Δ::TRP1 (K. lactis) spo12Δ::KanMX4 mam1:HIS3MX6*	3a and b
Y2152	MATa/α *spo11Δ::TRP1 (K. lactis) spo12Δ::KanMX4 cdc55Δ::CDC55-hphMX6::natMX4*	3b and 5
Y2153	MATa/α *spo11Δ::TRP1 (K. lactis) spo12Δ::KanMX4 cdc55Δ:: cdc55-MP-hphMX6 ::natMX4*	3b and 5
Y2157	MATa/α *spo11Δ::TRP1 (K. lactis) spo12Δ::KanMX4 cdc55Δ::cdc55-T5-hphMX6::natMX4*	3b
Y2424	MATa/α *spo11Δ::TRP1 (K. lactis) spo12Δ::KanMX4 cdc55Δ::cdc55-MP-hphMX6::natMX4 net1Δ::his5 net1-*	3b

	6CDK-TEVmyc9::TRP1	
Y1887	MATa/α *cdc55:: CDC55-hphMX6::natMX4 spo11:TRP1 spo12: KanMX4 P_{URA3}::tetR::GFP-LEU2 rec8:REC8-ha3::URA3 PDS1-myc18::TRP1(K.lactis) ura3/ura3:tetOx224:URA3*	4a
Y1896	MATa/α *cdc55:: cdc55-MP-hphMX6::natMX4 spo11:TRP1spo12: KanMX4 P_{URA3}::tetR::GFP-LEU2 rec8:REC8-ha3::URA3 PDS1-myc18::TRP1(K.lactis), ura3/ura3:tetOx224:URA3*	4a
Y3153	MATa/α *cdc55:: CDC55-hphMX6::natMX4 P_{URA3}::tetR::GFP-LEU2 rec8:REC8-ha3::URA3 PDS1-myc18::TRP1(K.lactis) ura3:tetOx224:URA3*	4b
Y3154	MATa/α *cdc55:: cdc55-MP-hphMX6::natMX4 P_{URA3}::tetR::GFP-LEU2 rec8:REC8-ha3::URA3 PDS1-myc18::TRP1(K.lactis) ura3:tetOx224:URA3*	4b
Y3153	*MATa/α cdc55::CDC55-hphMX6::natMX4 P_{URA3}::tetR::GFP::LEU2,rec8:REC8-ha3::URA3, PDS1-myc18::TRP1(K.lactis) ura3/ura3:tetOx224-URA3*	4c and d
Y3154	*MATa/α cdc55:: cdc55-MP-hphMX6::natMX4 P_{URA3}::tetR::GFP::LEU2,rec8:REC8-ha3::URA3 PDS1-myc18::TRP1(K.lactis) ura3/ura3:tetOx224-URA3*	4c and d
Y2421	MATa/α *spo11Δ::TRP1 (K. lactis) spo12Δ::KanMX4 cdc55Δ:: cdc55-MP-hphMX6:: natMX4 mad2Δ::HIS3MX6*	5
Y2485	MATa/α *cdc55::cdc55-MP-hphMX6::natMX4 spo11Δ::TRP1 (K.lactis) spo12Δ::KanMX4*	5

	clb3Δ::natMX4	
Y2578	MATa/α *spo11Δ::TRP1 (K. lactis) spo12Δ::KanMX4*<br><br>*CDC55/ cdc55:: cdc55-MP::hphMX6::natMX4*	5
